# Alternative Splicing under Cold Stress in Paper Mulberry

**DOI:** 10.3390/plants12233950

**Published:** 2023-11-23

**Authors:** Zhipeng Yu, Xia Huang, Shuhan Wen, Haijuan Cao, Nan Wang, Shihua Shen, Mingquan Ding

**Affiliations:** 1 The Key Laboratory for Quality Improvement of Agricultural Products of Zhejiang Province, College of Advanced Agricultural Sciences, Zhejiang A&F University, Linan, Hangzhou 311300, China; 13663997428@163.com (Z.Y.); 17318820085@163.com (X.H.); 2021601022025@stu.zafu.edu.cn (S.W.); chj102920@163.com (H.C.); jerrywang1010@sina.com (N.W.); 2 State Key Laboratory of Plant Diversity and Specialty Crops, Institute of Botany, Chinese Academy of Sciences, Beijing 100093, China

**Keywords:** paper mulberry, alternative splicing, differentially spliced genes, cold stress, DNA methylation

## Abstract

The paper mulberry is a commonly found tree species with a long history of cultivation. It also serves as a crucial case study for understanding how woody plants adapt to low temperatures. Under cold treatment, we observed a substantial number of alternative splicing (AS) genes, showcasing the intricate landscape of AS events. We have detected all seven types of AS events, with the alternative 3′ splice site (A3) having the most. We observed that many genes that underwent differential AS were significantly enriched in starch and sucrose metabolism and circadian rhythm pathways. Moreover, a considerable proportion of differentially spliced genes (DSGs) also showed differential expression, with 20.38% and 25.65% under 12 h and 24 h cold treatments, respectively. This suggests a coordinated regulation between gene AS and expression, playing a pivotal role in the paper mulberry’s adaptation to cold stress. We further investigated the regulatory mechanisms of AS, identifying 41 serine/arginine-rich (SR) splicing factors, among which 11 showed differential expression under cold treatment, while 29 underwent alternative splicing. Additionally, genes undergoing AS displayed significantly higher DNA methylation levels under cold stress, while normal splicing (non-AS) genes exhibited relatively lower methylation levels. These findings suggest that methylation may play an important role in governing gene AS. Finally, our research will provide useful information on the role of AS in the cold acclimation tolerance of the paper mulberry.

## 1. Introduction

The environment exerts various influences on plants, encompassing both high and low temperatures, exposure to UV radiation, salinity, droughts, flooding, mineral toxicity, and susceptibility to diseases [1]. Among these factors, cold stress stands out as a principal abiotic element that has detrimental effects on the growth and development of higher plants. It not only restricts the geographic distribution of plant species but also leads to reduced agricultural yields on a global scale [2]. Plants have developed intricate mechanisms to combat cold stress, encompassing components like signaling molecules, pathways for signal transduction, metabolic adjustments in response to cold stress, and interactions between regulatory pathways and other abiotic stress factors in plants [3]. An essential process within this context involves the alternative splicing (AS) of precursor mRNA (pre-mRNA) as a response to fluctuations in temperature [4,5,6]. Typically, in plants, there are seven fundamental types of AS events that occur based on the choice of splice sites within the same pre-mRNA molecule. The options include retained intron (RI), skipping exon (SE), mutually excluding exons (MX), alternative first exon (AF), or alternative last exon (AL) when splice site selection is omitted. Alternatively, when distinct splice sites are chosen, this leads to the creation of the alternative 5′ splice site (A5) and alternative 3′ splice site (A3) [7].

AS is a pivotal regulatory mechanism that significantly augments cellular and functional complexity. It has garnered extensive attention in both the animal and plant realms [8,9]. A substantial body of evidence suggests that plants harness AS as an adaptive strategy to cope with various abiotic stresses [8,10,11]. In the context of temperature signaling pathways in plants, AS is recognized as a mechanism for sensing temperature fluctuations and potentially fine-tuning the coding sequence of genes responsive to cold stress. The responsiveness of AS to cold conditions is notably swift, occurring within minutes [12,13,14,15]. It is estimated that approximately 33% of transcripts involved in the cold stress response undergo AS [16]. Recent research has unveiled extensive and rapid AS changes in reaction to cold stress. Hundreds of genes, including cold-responsive transcription factors and serine/arginine-rich (SR) splicing factors/RNA-binding proteins, exhibit alterations in their expression levels due to the swift occurrence of AS events [12]. Furthermore, AS serves as a critical link between the regulation of gene expression and mechanisms like peptide interference (PEPi) and/or nonsense-mediated decay (NMD) [17]. In the face of cold stress, transcripts of *DREB* genes undergo AS, where temperature fluctuations regulate the abundance of specific isoforms. For example, in wheat, the *WDREB2* gene, sharing homology with *Arabidopsis thaliana DREB2A* and *DREB2B* genes, undergoes AS through exon skipping, yielding three splice variants at 4 °C [18]. Similarly, the rice *DREB2*-type gene *OsDREB2B* displays AS, producing two active variants, *OsDREB2B1* and *OsDREB2B2*, in response to low-temperature conditions [19]. In addition to *DREBs*, *COR* genes also undergo AS when exposed to cold stress. In the tea plant (*Camellia sinensis*), the *CsCOR* gene has been noted to undergo AS, resulting in the formation of a truncated protein at low temperatures. Nevertheless, it remains unreported whether these truncated proteins serve a specific biological function [15].

AS in plant genes is a complex and finely regulated process influenced by numerous molecular mechanisms, including splicing factors, splicing site recognition, epigenetic modifications, and the sequences within exons and introns [20]. AS occurs within the spliceosome, which comprises small nuclear ribonucleoproteins (snRNPs) and a multitude of Ser/Arg-rich proteins [4,21,22,23]. Ser/Arg-rich proteins, exhibiting high conservation in plants, function as SRs in both constitutive and AS, guiding the selection of splice sites through the formation of distinct spliceosome complexes [24,25]. In *Arabidopsis thaliana*, the expression and the splicing patterns of the SR genes are differentially regulated by low-temperature stress [26]. In the woody plant *Populus trichocarpa*, it was shown that cold stress significantly altered the expression and splicing patterns of 18 *PtSR* genes, which made up around 75% of the total SR family genes. This alteration, in contrast to their steady expression under normal development settings, suggests the possible regulatory involvement of *PtSR* genes in stress responses, especially under abiotic and hormonal challenges, notably cold stress [27].

The discovery that a substantial portion of pre-mRNA splicing often occurs concurrently with transcription indicates the potential involvement of epigenetic mechanisms in the regulation of splicing [28]. Epigenetic modifications can influence both chromatin structure and the rate of RNA polymerase II (Pol II) elongation, thus having the capacity to directly or indirectly recruit spliceosomal proteins [29,30,31]. Among the various forms of epigenetic modifications, DNA methylation in the CpG context stands out as a prominent player present in most eukaryotes [32]. Studies have revealed that DNA methylation exerts a widespread impact on AS, especially concerning the splicing of alternative exons. The reduction in CpG methylation levels is linked to a noteworthy alteration in the ratio of AS [29,33]. Nevertheless, our current knowledge regarding whether and to what extent DNA methylation might impact AS in plants remains limited [34].

The paper mulberry (*Broussonetia papyrifera*) is a common local tree species with a long history of cultivation. It has good ecological and economic benefits. It is a potential substance for understanding how woody plants adapt to cold temperatures because of its high cold resistance [35]. Early research on the cold resistance of the paper mulberry has revealed that initial exposure to cold stress leads to a decrease in respiratory metabolism and a reduction in the transport and hydrolysis of photosynthetic products, resulting in starch accumulation within chloroplasts. Concurrently, it influences signal transduction, stress defense mechanisms, and secondary metabolic pathways, while inhibiting photosynthesis [36]. AP2/ERF, bHLH, MYB, NAC, WRKY, ARF, DBB, G2-like, GRF, GRAS, LBD, WOX, and YAABY are only a few of the TF families involved in controlling growth and development that show significant potential growth regulatory capabilities under cold stress [35]. The gene family *PP2C* in the paper mulberry takes has a role in the control of the cold stress response. Three *BpPP2C* proteins (Bp01g0320, Bp01g0512, Bp09g1278) are crucial, while Bp01g0512 and Bp09g1278 are important for the ABA signaling pathway and the *MAPK* cascade, respectively [37]. According to studies, the paper mulberry’s specific cold signaling transduction and controlled pathways are mediated by *LRR-RLK*, *BCS1 ATPase*, and *PP2A* [38]. Currently, most cold stress research is concentrated at the transcriptome level. Nonetheless, there is presently limited knowledge regarding its capacity to endure low temperatures at the level of AS. Given the accessibility of the complete genome sequences of the paper mulberry, we are now equipped to utilize AS analysis utilizing RNA-seq data. This approach enables us to probe into the molecular mechanisms governing the response and regulation of cold-stressed paper mulberry in a new direction.

In this study, our primary objective was to conduct a thorough investigation into the molecular response of the paper mulberry when subjected to cold stress, specifically focusing on the realm of AS. To accomplish this, we harnessed the power of RNA-Seq analysis, examining leaves exposed to both cold and standard environmental conditions. Our goal was to not only identify AS isoforms but also quantify the differential alternative splicing (DAS) events occurring in response to cold stress on a whole genome scale. This comprehensive analysis revealed an astonishing 7841 AS genes, encompassing approximately 23,000 AS events, shedding light on the multitude of splicing changes incited by cold stress. By integrating DNA methylation data, we not only improved the annotation of the paper mulberry’s transcriptome but also provided an initial glimpse into the intricate mechanism governing AS in the face of cold stress. These findings underscore the fundamental role of AS in the paper mulberry’s response to cold stress and serve as a valuable reference point for understanding similar responses in other woody plant species.

## 2. Results

### 2.1. Analysis of AS Genes throughout the Paper Mulberry Genome

In order to investigate the AS genes and AS events in the transcriptome of the paper mulberry under cold stress, we subjected its seedlings to 12 h and 24 h cold stress treatments, respectively. For the control and cold stress-treated samples, RNA sequencing yielded a total of 54 G short reads. Then, in order to obtain the most complete transcript information, we rebuilt the transcripts. When combined with the original gene annotation of the paper mulberry genome, a total of 31,524 genes and 3746 new genes were obtained, of which 24,285 genes on average were expressed in the leaves.

**Figure 1:**
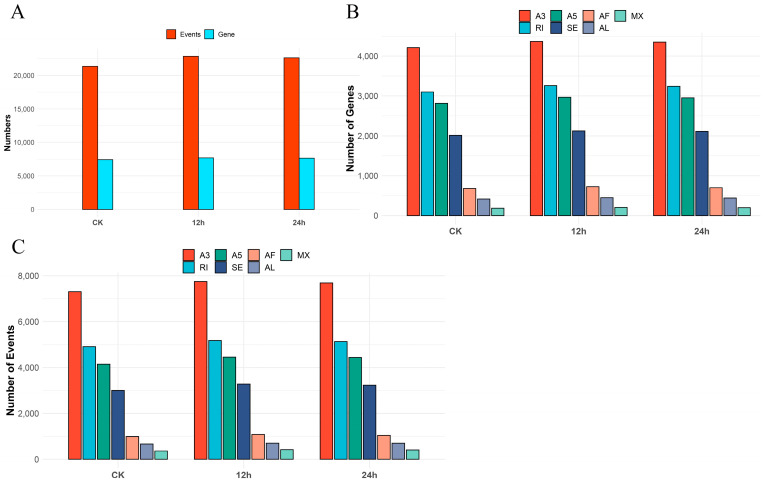
The statistical analysis of AS genes and events in samples subjected to both CK (control) and cold treatment conditions. (**A**) The bar plot of the counts of AS genes and AS events in various samples. (**B**) The bar plot of the counts of AS genes categorized by different AS types. (**C**) The bar plot of the counts of AS events categorized by different types of AS. A5: Alternative 5′ splice site; A3: Alternative 3′ splice site; AF: Alternative first exon; MX: Mutually exclusive exons; RI: Retained intron; SE: Skipping exon; AL: Alternative last exon.

In total, 7841 genes were found to be alternatively spliced, resulting in a total of 23,762 events, according to an examination of AS profiles. Of these, 7438 genes underwent AS and generated 21,380 events in the CK group, 7705 genes underwent AS and generated 22,864 events in the 12 h cold-treated group, and 7664 genes underwent AS and generated 22,637 events in the 24 h cold-treated group (Figure 1A, Supplemental Table S1).

We detected 3328 genes out of all the expressed genes that had only one AS event, which accounted for 10.55% of the total; 4513 genes had more than one AS event, which accounted for 14.32%; and 23,683 genes were found to have no AS in any of the samples, which accounted for 75.13% of the total (Supplemental Table S2). On average, there were 3.03 alternative splicing events per gene. Genes with the A3 splicing type were the most common among all types (Figure 1B). The splicing type distribution analysis indicates that the CK and cold stress groups are similar and that A3 had the most AS events (7302, 7750, 7689) followed by A5 (4143, 4451, 4436), RI (4912, 5175, 5128), SE (2999, 3277, 3227), AF (990, 1080, 1040), AL (669, 705, 704), and MX (365, 426, 413) (Figure 1C).

### 2.2. Identification of Differentially Spliced Genes in Paper Mulberry under Cold Stress

**Figure 2:**
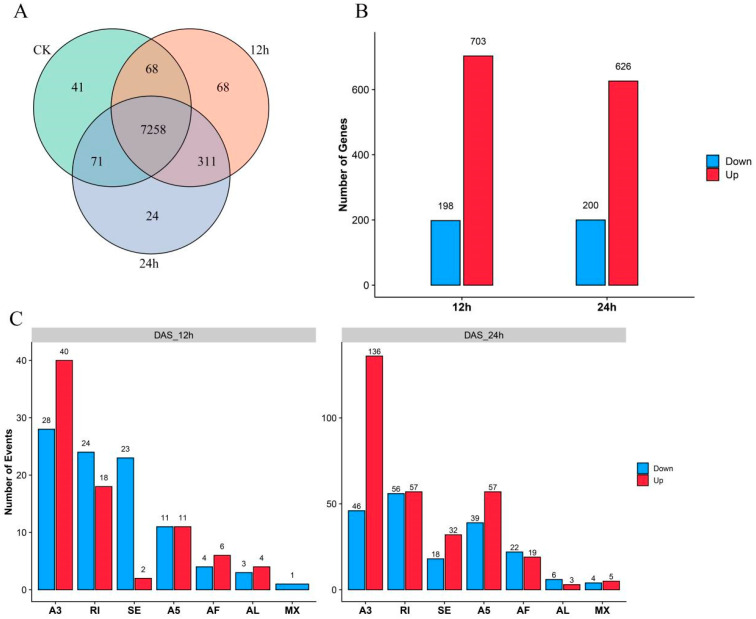
The statistical analysis of the differentially spliced genes (DSGs) and differential alternative splicing (DAS) events. (**A**) The Venn diagram of specific AS genes (SAS) at three time points. (**B**) The barplot of number-altered splicing gene (NAS) counts at 12 h and 24 h cold treatments. (**C**) The barplot of various types of DAS events at 12 h and 24 h.

We found that AS genes in the leaves remained constant during cold stress. Over 7258 of these genes were alternatively spliced in the CK, 12 h, and 24 h groups. Furthermore, we identified genes (SAS) that are specifically prone to AS in each group: 41 genes are particular to CK, 68 genes are specific to cold treatment for 12 h, and 68 genes are specific to 24 h cold treatment. (Figure 2A). In addition to SAS, we examined genes (NAS) with AS event number variations. Taking CK as a reference, we found that 901 genes have more than two-fold changes in the number of AS events relative to CK at 12 h cold treatment, and 826 genes at 24 h (Figure 2B). This category encompasses genes that displayed AS events in one treatment but not in the other. We also focused on the genes (DAS) that showed significantly differential expression changes in AS isoforms under cold stress (Supplemental Table S3). Compared with CK, there were 175 DAS events in the 12 h treatment, and there were 500 DAS events in the 24 h treatment (Figure 2C). In total, we identified 1040 differentially spliced genes (DSGs) during the 12 h cold treatment and 1232 DSGs during the 24 h cold treatment.

**Figure 3:**
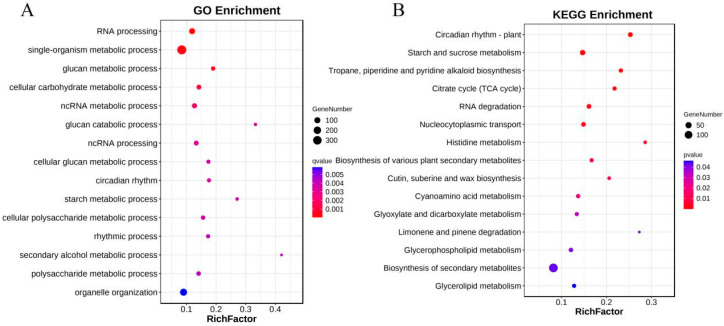
GO and KEGG enrichment analyses for DSGs. (**A**) Bubble chart illustrating the results of GO enrichment analysis for DSGs. (**B**) Bubble chart visualizing the outcomes of KEGG enrichment analysis for DSGs.

To study the functions of these differentially spliced genes (DSGs), including SAS, NAS, and DAS genes. Under cold stress, we performed Gene Ontology (GO) analysis and Kyoto Encyclopedia of Genes and Genomes (KEGG) analysis on the union of the three gene sets. GO analysis showed that 73 GO terms were significantly enriched under cold stress, among which the top 15 significantly enriched GO terms mainly included genes in categories such as RNA processing, ncRNA processing, glucan metabolic, carbohydrate metabolism, starch metabolism, polysaccharide metabolism, rhythmic process, circadian rhythmic process and so on (Supplemental Table S4). KEGG analysis showed that sixteen pathways were significantly enriched, among which the top five significantly enriched pathways mainly included circadian rhythm, starch and sucrose metabolism, tropane, piperidine and pyridine alkaloid biosynthesis, citrate cycle (TCA), and RNA degradation (Supplemental Table S5). Both GO and KEGG analyses indicated that GO terms and pathways related to circadian rhythm and carbohydrate metabolism were significantly enriched (Figure 3A,B).

**Figure 4:**
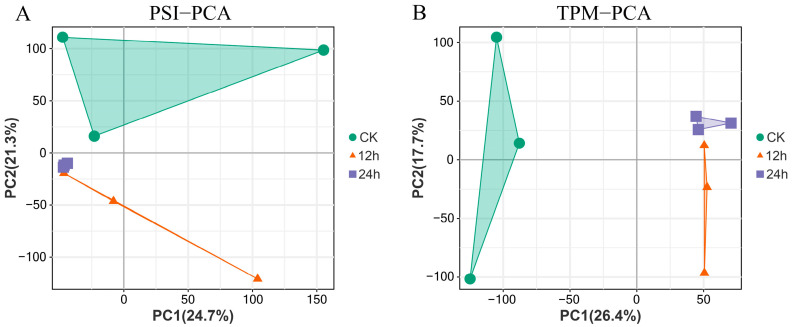
Principal Component Analysis of AS genes and transcriptome data. (**A**) The PCA analysis outcomes using PSI values, displaying distinct clustering of CK (green circles), 12 h (blue squares), and 24 h (orange triangles) samples. (**B**) The PCA analysis based on the TPM values.

We then performed PCA analysis on the percent spliced in index (PSI) values of each gene in order to investigate the regulatory role of AS in the cold stress of the paper mulberry. The study discovered that CK, 12 h, and 24 h cold treatments were all clustered in separate groups and that the PSI clustering results of splicing genes were compatible with the clustering results of gene expression (Figure 4A,B). This shows that AS genes are likely consistent with gene expression in response to cold stress. In light of this, we further contrasted the association between AS genes and genes that exhibit differential expression in response to cold stress.

### 2.3. The Regulation of AS in Paper Mulberry

**Figure 5:**
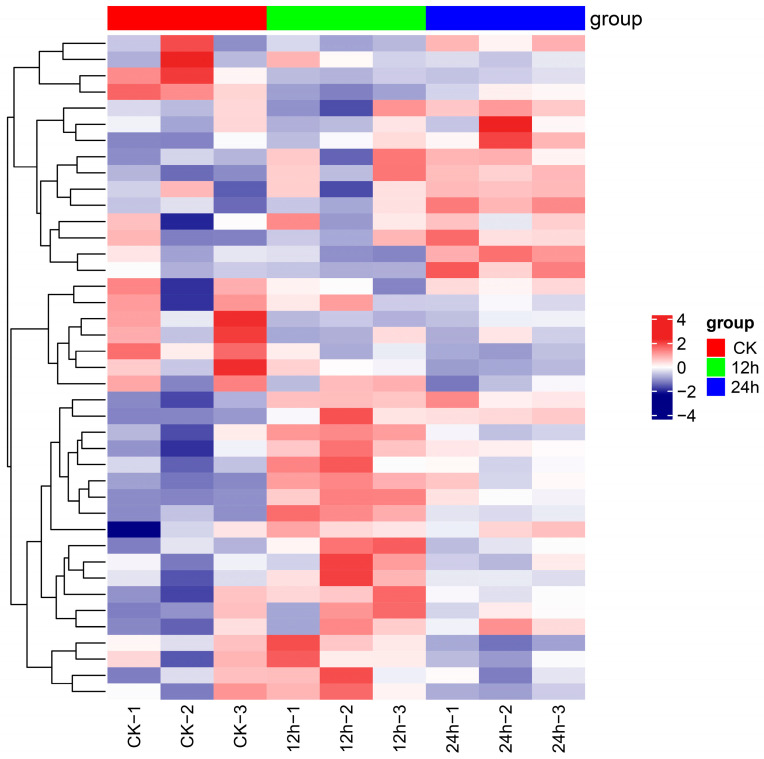
Heatmap depicting the expression levels of SR genes in CK, 12 h, and 24 h cold-treated samples.

We analyzed the regulatory systems governing gene AS of the paper mulberry in order to understand the fundamental causes of this phenomenon. Initially, we assessed the expression levels of SR genes, which have been widely recognized as pivotal regulators of alternative gene splicing in plants. A total of 41 SR genes were identified, of which 29 underwent alternative splicing (Supplemental Table S6). Upon investigating the expression of these SR genes in response to cold treatment durations of 12 and 24 h, we observed that a total of 11 SR genes exhibited differential expression during the cold treatment. Specifically, following a 12 h cold treatment, seven SR genes displayed altered expression, with four of them being up-regulated and three down-regulated. Meanwhile, in the case of a 24 h cold treatment, nine SR genes exhibited variable expression, with five of them showing increased expression and four showing decreased expression (Figure 5). Additionally, five SR genes showed differential expression in both the 12 h and 24 h treatments.

**Figure 6:**
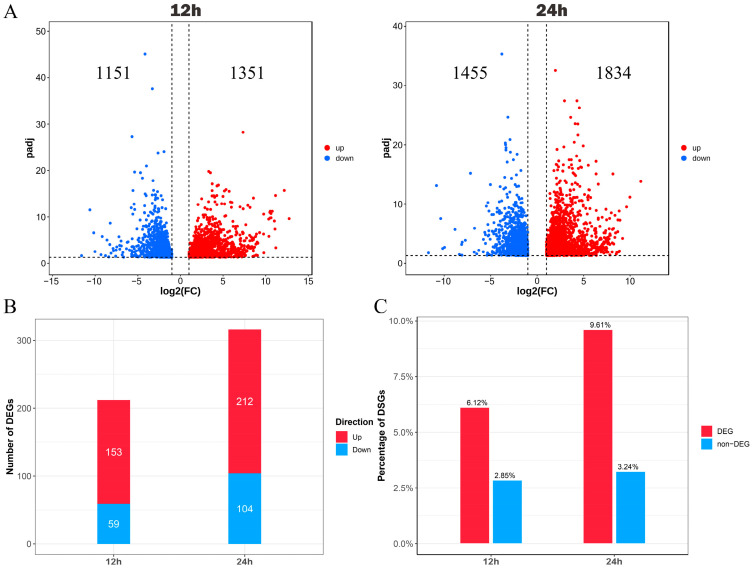
The investigation of the connection between DEGs and DSGs in the context of cold stress at 12 h and 24 h. (**A**) Volcano plot highlighting up-regulated and down-regulated genes following 12 h and 24 h cold treatments. (**B**) The barplot illustrating the count of up-regulated and down-regulated DEGs within the DSGs. (**C**) The percentage representation of DSGs within both DEGs and non-DEGs.

We integrated the analyses of the DSGs and differentially expressed genes (DEGs) under 12 h and 24 h cold treatments in order to better understand the association between transcriptome regulation and AS under cold stress. There were a total of 1040 DSGs discovered after 12 h of cold treatment. Among them, we identified 212 DEGs, accounting for 20.38% of all DSGs, wherein 153 genes showed up-regulation and 59 genes showed down-regulation. After being exposed to cold for 24 h, 1232 DSGs were noted. Additionally, we identified that more DSG expression patterns were altered and we discovered 316 DSGs with differential expression patterns, accounting for 25.65% of all DSGs (Figure 6B). Among them, 104 genes were down-regulated and 212 genes were up-regulated. We examined the changes in the gene expression pattern in these DSGs to see whether AS is correlated with DEGs. The fraction of DSGs that were up- or down-regulated under the 12 h and 24 h cold treatments differed significantly. Our finding suggested that AS was correlated with more up-regulated than down-regulated DEGs in our investigation.

Under 12 h and 24 h of cold treatment, there were 2502 and 3289 genes differentially expressed, respectively (Figure 6A). We analyzed the frequency of DSGs in the DEGs and non-differentially expressed genes (non-DEGs) in order to investigate whether transcriptional activity is associated with AS alterations in the paper mulberry under the 12 h and 24 h cold treatments. According to our results, under the 12 h cold treatment, the proportion of DSGs in DEGs was substantially larger than that in non-DEGs (6.12% vs. 2.85%). Meanwhile, under the 24 h cold treatment, the proportion of DSGs in DEGs was substantially larger than that in non-DEGs (9.61% vs. 3.24%). This finding is consistent with the hypothesis that transcriptional activity and AS activity may indeed be connected (Figure 6C).

**Figure 7:**
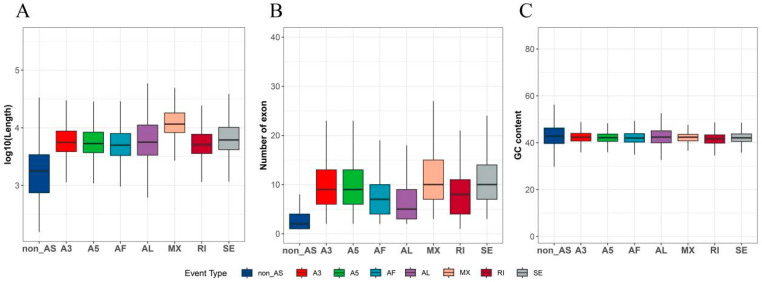
Characteristics of transcripts undergoing different types of AS events, encompassing their length distribution, exon count, and GC content. (**A**) Length analysis for various types of AS transcripts. (**B**) Examination of the exon count for different types of transcripts in AS genes. (**C**) Evaluation of the GC content among different types of AS transcripts.

In addition to investigating the relationship between DEGs and AS genes, we employed statistical analysis techniques to examine whether sequence composition attributes of AS transcripts, such as GC content, transcript length, the number of exons, etc., are associated with gene AS within the paper mulberry genome. This study revealed that, in comparison to normally spliced transcripts, the transcripts of AS genes exhibit significantly longer gene lengths and a notably higher number of exons, while the GC content remains unchanged (Figure 7A–C).

### 2.4. A Comprehensive Analysis of DNA Methylation and Alternative Splicing in Paper Mulberry under Cold Stress

**Figure 8:**
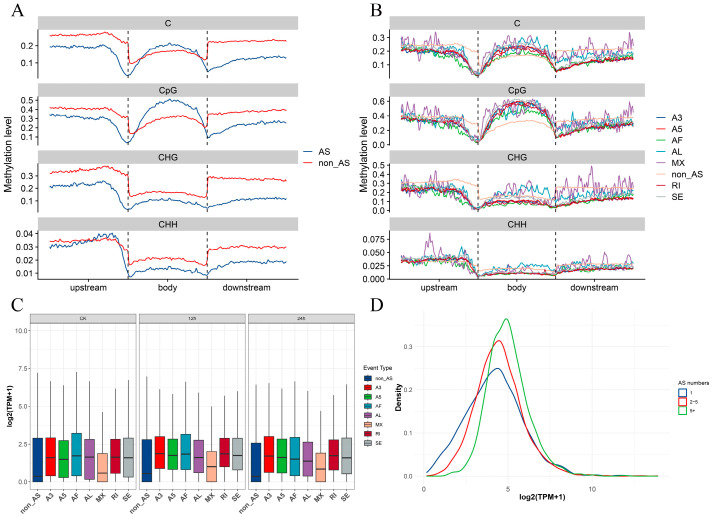
Exploration of DNA methylation and transcriptional patterns in AS genes. (**A**) Methylation level distributions in AS genes compared to normal splicing (non-AS) genes. (**B**) Methylation level distributions among different AS types. (**C**) Box plot representing the transcriptional levels of different AS types. (**D**) Density plot depicting transcriptional levels in genes with varying quantities of AS events.

We expanded our analysis to examine the methylome of genes that undergo AS, aiming to explore the potential influences of epigenetic modifications on these genes. The results highlight that within the gene body regions, genes undergoing AS display notably higher levels of m5C DNA methylation, especially in relation to CpG, when compared to non-splicing genes (Figure 8A). This pattern is also similarly observed in various types of alternative splicing transcripts (Figure 8B). Conversely, in the upstream transcription initiation sites, the DNA methylation levels of AS genes are significantly lower than those observed in their non-splicing genes. In plants, there is typically a positive correlation between the DNA methylation levels within the gene body and gene expression. As a result, we formulated the hypothesis that the transcription levels of transcripts undergoing AS would be relatively higher compared to those in non-splicing genes. Our analysis of the transcriptome data confirms that all types of the transcripts of AS genes indeed exhibit higher expression levels than normally spliced genes and the expression level of genes with more AS events is higher than those with fewer AS events (Figure 8C,D). Consequently, our data provide evidence supporting the idea that AS transcripts are regulated by a combination of factors, including their elevated transcript levels, which are influenced by lower methylation levels at their transcript initiation sites and higher methylation in their transcript bodies.

To look into any possible relationship between DNA methylation and DAS in cold-stressed paper mulberry, we conducted an analysis to identify overlapping genes between differentially methylated genes (DMGs) and DAS genes (Supplemental Tables S7 and S8). Our findings reveal that, following 12 h and 24 h cold treatments, there are zero and four DAS genes that overlap with DMGs, constituting 0% and 0.78% of all DAS genes, respectively. However, the relatively limited number of overlapping genes suggests that alterations in methylation status may not be closely linked to the changes observed in DAS events induced by cold treatments of the paper mulberry.

## 3. Discussion

AS is recognized as a primary mechanism influencing protein diversity during both plant growth and responses to stress [5]. Numerous studies have highlighted that developmental growth in woody plants involves significant alterations in gene expression patterns driven by alternatively spliced transcripts. Moreover, woody plants exhibit substantial variations in AS among functionally relevant genes to effectively respond to various stressors [6]. Nonetheless, comprehensive investigations into the genome-wide AS of genes in woody plants under stress conditions have been limited, potentially attributed to the absence of chromosome-scale whole-genome sequences. The paper mulberry is well known for its outstanding ability to withstand cold conditions at −30 °C [35]. With access to the complete genome sequence, prior studies have identified genes responsive to cold stress in the paper mulberry by using transcriptome analysis [38]. Nonetheless, the extent to which AS contributes to the paper mulberry’s cold resistance remains uncertain. The present study’s identification of genome-wide AS events in response to cold stress provides new insights into the role of AS in facilitating the paper mulberry’s adaptation to low temperatures.

Various types of AS events can be generated by AS genes [39]. In woody plants like the tea plant (*Camellia sinensis*) and *Populus trichocarpa*, previous research has indicated that RI is the dominant form of AS. However, our investigations have revealed that the prevailing type is A3, even though AS genes of the RI type constitute a significant proportion of the total AS genes. AS events of the A3, RI, and A5 types often lead to the production of truncated proteins, introducing premature termination codons (PTCs) into spliced transcripts, which hinder the generation of functional proteins [40]. The abundance of A3, RI, and A5 genes induced by cold stress suggests that cold stress can trigger a substantial number of genes to undergo the NMD pathway, resulting in the degradation of genes that are already actively transcribed.

Plants respond to abiotic stress with a substantial occurrence of AS events [41]. For instance, during cold acclimation in tea plants, AS events are evident in more than 41% of genes [15]. In cassava (*Manihot esculenta Crantz*), approximately 31.6% of protein-coding genes have been observed to undergo AS events under cold stress [42]. Our research has similarly found that a significant portion of genes, approximately 24.7%, undergo AS in the paper mulberry in response to cold stress. Additionally, we found that under 12 h of cold treatment, 20.38% of DSGs showed differential expression, while 25.65% of DSGs showed differential expression under 24 h of cold treatment. This is consistent with the idea that the paper mulberry’s ability to adjust to cold stress is possibly influenced by the coordinated regulation of gene AS and expression. In the future, in order to confirm whether these modifications can improve the paper mulberry’s resistance to cold stress, we will investigate these genes more thoroughly and carry out experiments on gene functions by using gene overexpression or gene editing.

Under cold stress, many functional genes undergo AS. A study on *Arabidopsis thaliana*, for instance, revealed a significant increase in sugar metabolism-related pathways during cold acclimation [12]. In our investigation, we conducted GO and KEGG analysis of AS genes (SAS, NAS, DAS) and found that GO terms and pathways linked to carbohydrate metabolism were notably enriched. Consequently, we infer that changes in the quantity and expression of AS can regulate sugar metabolism, thereby enhancing the low-temperature tolerance of the paper mulberry. Previous research has identified the AP2/ERF, bHLH, MYB, NAC, and WRKY gene families as pivotal players in the cold stress response of the paper mulberry [38]. In our study, we also discovered that numerous genes from these transcription factor families undergo AS in response to cold stress. Notably, some of these genes, such as *bHLH* (Bp07g0805, Bp04g1148), *MYB* (Bp06g0978, Bp08g0394), *ERF* (Bp13g1306, Bp13g1307), *WRKY* (Bp07g0107, Bp07g0679), and *NAC* (Bp01g3211), were found to undergo AS during cold treatment. It is worth noting that our study also revealed genes previously mentioned in the Genome-Wide Association Study (GWAS) related to cold stress, including LRR receptor-like serine threonine-protein kinase (Bp06g1029, Bp06g1443), polyadenylate-binding protein-interacting protein (Bp08g0526, Bp01g1842), AAA-type ATPase family protein (Bp11g1094), E3 ubiquitin-protein ligase (Bp08g0224, Bp06g1537), and ethylene-responsive transcription factors (Bp07g1222, Bp11g1327), were identified as undergoing AS in response to cold treatment. This is consistent with the hypothesis that these genes may also play a crucial role in enhancing the paper mulberry’s cold resistance when exposed to low-temperature conditions through the modulation of AS.

Temperature-related AS events control plant biological clocks as well. The circadian clock adjustment factors include circadian clock-associated 1 (*CCA1*), late elongated hypocotyl (*LHY*), pseudo-response regulator (*PRR5, PRR9*, *PRR7*), and timing of cab (*TOC1*). Studies have shown that the Arabidopsis *LHY* transcript was affected by temperature variations and that AS in *LHY* is temperature-dependent [43,44]. We have also found this phenomenon in woody plants. Five types of LHY’s isoforms (A3, A5, MX, RI, and SE) were discovered at 3 °C in a study on *Populus trichocarpa* [44]. In this study, we also found that many clock genes undergo AS, such as *LHY* (Bp01g0716), *PRR5* (Bp03g0177), *PRR7* (Bp03g1216, Bp09g1375, Bp03g1217) and so on, proving that circadian clock genes undergo AS to regulate plant tolerance under cold stress. The results should be further confirmed by more detailed time series experiments with corresponding time point controls to explore how photoperiod genes change under cold stress. In addition, the response mechanism to cold stress through AS mediated by clock genes in woody plants remains unclear, and further functional studies are required to confirm our findings.

Research has provided evidence that AS can be regulated not only by SR genes, but also by factors such as length, the number of exons/introns, and the GC content of AS genes [20,27]. Additionally, epigenetic regulation such as DNA methylation levels has been shown to influence AS [34]. For instance, SR genes, which are essential regulators of plant AS, respond to a range of temperatures. In *Populus trichocarpa*, approximately 83% of *PtSR* genes react to various stresses, especially cold stress, which significantly affects the expression and AS of about 75% of these genes [27]. In our study, when comparing the SR gene series of *Populus trichocarpa* to the paper mulberry, we identified a total of 41 SR genes, with 29 of them undergoing AS under cold treatment, constituting roughly 70%. Thus, the serine/arginine-rich proteins can play a crucial role in regulating plant AS in the paper mulberry. Furthermore, we noticed that AS is more prevalent in transcripts with longer sequences and more exons. Similar results have been reported in cotton (*Gossypium hirsutum*) [20], as well as in soybeans [43]. This suggests that the gene structure and sequence composition in woody plants, much like in crops, have a similar influence on AS, potentially indicating a conserved phenomenon. DNA methylation, a significant epigenetic modification, has diverse effects on biological processes and plays a crucial role in plant responses to cold stress [15]. However, a limited correlation between DMGs and DAS has been documented. The mechanisms through which DNA methylation regulates AS are still not fully understood. In walnuts (*Juglans regia*), AS-related genes exhibit higher methylation levels than non-AS genes in all three contexts (CpG, CHG, and CHH) within the gene body [45]. However, in the case of the paper mulberry, we observed a different pattern. In the CpG context, the methylation levels were consistent with walnuts, with AS genes showing higher methylation levels. In contrast, in the CHH and CHG contexts, the results were reversed, with normal spliced genes exhibiting higher methylation levels compared to AS genes.

In this study, we primarily focused on leaf tissue subjected to short-term (12, 24 h) cold stress; we did not consider the influence of season-related factors. Additionally, we only used leaves from the seedling stage for experiments and analysis. Research involving roots, bark, and long-term cold stress remains unexplored in our investigation. Furthermore, considering the spatial and temporal specificity of gene expression, the AS information we detected may not be exhaustive. In the future, several studies should be conducted to overcome the shortcomings. For example, more detailed time series experiments and reasonable controls should be set to explore the response of photoperiod-related genes to cold stress. Secondly, more material tissues such as bark and roots should be included for long-term cold stress. Thirdly, with the cost drop of Iso-seq, we plan to incorporate third-generation sequencing technologies to comprehensively profile the entire plant transcriptome, allowing for a more in-depth exploration of the AS mechanisms in woody plants during cold stress.

## 4. Materials and Methods

### 4.1. Plant Materials

We planted paper mulberry seeds in growth chambers at 26 °C, a 14/10 h photoperiod (day/night), and humidity of 70% RH [38]. Seeds were grown in flower pots with a bottom diameter of 5 cm, a diameter of 7 cm, and a height of 7.3 cm, using a 1:1 ratio of nutrient soil and vermiculite for planting. Two-month-old seedlings of paper mulberry were used for the research. The cold experiment was conducted in a growth chamber with the same conditions and a temperature of 4 °C. The entire two-month-old seedlings used for cold treatments were placed in the 4 °C growth chamber. For these experiments, we used leaves from paper mulberries that had not undergone cold treatment as a control group. During the cold stress application, leaves were harvested at two time points, 12 h and 24 h of cold exposure. This study employed a mixed sampling strategy to eliminate inter-individual variations. Three seedlings were combined into one sample, serving as a biological replicate. The collected samples were rapidly frozen in liquid nitrogen and subsequently stored at −80 °C to ensure preservation. These samples were later utilized for DNA methylation and transcriptome sequencing analysis.

### 4.2. Transcriptome Sequencing

We utilized the Agilent 2100 bioanalyzer to assess the integrity and total amount of RNA. Starting with total RNA, we enriched mRNA with polyA tails using Oligo(dT) magnetic beads and subsequently fragmented it. Through a series of procedures, we successfully constructed a cDNA library with a size range of 370–420 bp. Upon verification with Qubit2.0 and Agilent 2100, the library was sequenced on the Illumina platform, producing 150 bp paired-end reads. This sequencing approach is based on the “sequencing by synthesis” principle, ensuring data quality [46].

### 4.3. RNA-Seq Data Analysis

Initially, we employed the fastp software (version: 0.23.2) [47] to quality-control and filter the sequencing reads obtained. Subsequently, the purified paired-end sequencing reads were aligned to the reference genome of paper mulberry using hisat2 (version: 2.2.1) [48]. To facilitate further analysis, we converted the genome annotation files of the reference genome into gtf format using the Gffread software (version: 0.12.7) [49]. Using the Rsubread package (version: 2.12.3) in R [50], we calculated the read counts and corresponding TPM values for each gene. In addition, the StringTie software (version: 2.2.1) [51] was utilized to predict novel transcripts, which were then integrated with the original gtf annotation files of the reference genome. To achieve a comprehensive expression analysis of genes and transcripts, we used Salmon (version: 1.4.0) [52] to compute read counts and TPM values for all genes and each transcript. Finally, leveraging the Trinity software (version: 2.11.0) [53] and based on DESeq2 parameters, differential gene expression analysis was conducted between two comparison sets (12 h vs. CK and 24 h vs. CK). Genes with adjusted *p*-values < 0.05 (padj < 0.05) while the difference multiplier was satisfied ((log_2_(fold-change) ≥ 1) were considered as DEGs.

### 4.4. AS Analysis

We employed the suppa2 [54] for AS analysis. Initially, using the generateEvents option, we predicted all potential AS events. Subsequently, in conjunction with the TPM values for each transcript, we calculated the psi values for each event using the psiPerEvent option. When the PSI value of an AS event exceeded 0, we considered that the event had undergone AS. Consequently, the gene hosting this event was also classified as a gene exhibiting AS. To identify differential AS events, we applied the diffSplice option, based on the psi values of the events and the TPM values of the transcripts. We defined AS events with a change rate in PSI value (dPSI) greater than 0.3 and a *p*-value less than 0.05 as DAS events.

### 4.5. Functional Annotation and Enrichment Analysis for Paper Mulberry Genes

For functional annotation of genes from the paper mulberry, we utilized EggNOG and OmicsBox (https://www.biobam.com/omicsbox/) (accessed on 19 June 2023) software for GO annotation. KEGG pathway annotations were conducted using EggNOG (version: 2.1.11) [55] and KofamKOALA (version: 107.0) [56]. Further, differential AS genes were enriched for GO and KEGG pathways utilizing OmicShare (https://www.omicshare.com/).

### 4.6. Identification of SR Genes in Paper Mulberry

We initiated our analysis by conducting a multiple sequence alignment of SR from the *P. trichocarpa* genes [27] using MAFFT (version: 7.475) [57]. Subsequently, we employed HMMER (version: 3.3.2) [58] to align the genes of paper mulberry with those of SR genes in *P. trichocarpa*, employing an e-value threshold of 1 × 10^−20^. Following this alignment, we further scrutinized the identified paper mulberry SR genes by conducting searches in the NCBI database to confirm their status as SR.

### 4.7. Whole Genome Bisulfite Sequencing (WGBS)

Samples underwent DNA testing and were subsequently enhanced with positive control DNAs. These were then fragmented into 200–400 bp sizes using the Covaris S220. The ssDNA fragments, post-bisulfite treatment, saw unmethylated cytosines converted to uracils, leaving the methylated ones intact. The fragments were then processed through adapter ligation, size selection, and PCR amplification. Using the Accel-NGS Methyl-Seq DNA Library Kit, a low-complexity tail was appended to each fragment’s 3′ end. For optimal sequencing results, we trimmed 10 bases from the outset of each read to avoid potential methylation artifacts. After these steps, the library’s integrity was validated using the Agilent 5400 system, quantified at 1.5 nM via QPCR, and then pair-end sequenced on Illumina platforms.

### 4.8. BS-Seq Analysis

We employed the Bismark (version: 0.23.1) [59] software to align the sequencing data to the reference genome. Subsequently, the methylation levels at each site were computed using Batmeth2 [60]. A sliding window approach was utilized to determine the methylation level of the gene body region of each gene as well as the 2000 bp upstream and downstream regulatory regions (window size: 0.02 times the gene length or regulatory regions; step size: 0.01 times the gene length or regulatory regions). For the analysis of differentially methylated genes, we used the methylKit (version: 1.24.0) [61]. Genes with a methylation change rate (meth.diff) exceeding 0.2 and a q-value not exceeding 0.05 were classified as DMGs.

## 5. Conclusions and Future Work

In this study, we conducted a comprehensive analysis of AS events in the transcriptome of the paper mulberry under cold stress. AS plays a vital role in regulating key biological processes, including RNA processing, carbohydrate metabolism, and circadian rhythms. The occurrence of AS may be related to the length, the number of exons, and the expression level of transcripts. Most of the SR genes undergo AS and differential expression under cold stress. Additionally, we compared the DNA methylation levels between AS genes and non-AS genes, shedding light on potential epigenetic regulatory mechanisms. These findings provide a valuable foundation for future research into the mechanisms and functional implications of AS in plant stress responses.

This study provides valuable insights into the role of AS in the response of the paper mulberry to cold stress. However, there are still several avenues for further research and exploration. On the one hand, future investigations should include functional validation studies to confirm the roles of specific AS events and genes in cold stress adaptation. On the other hand, elucidating the precise molecular mechanisms and regulatory pathways governing AS under cold stress will provide a deeper understanding of plant responses to environmental challenges.

## Data Availability

All raw RNA-seq illumina paired-end reads of transcriptomes from this project have been submitted to the National Genomics Data Center (GSA accession number CRA013391): https://bigd.big.ac.cn/gsa/browse/CRA013391 (accessed on 20 November 2023).

## Supplementary Materials

The following supporting information can be downloaded at: https://www.mdpi.com/article/10.3390/plants12233950/s1, Table S1: Alternative splicing events occurring in samples of CK, 12 h, and 24 h; Table S2: Gene-Splicing Event Classification; Table S3: Different types of Differential Spliced Genes (DSG) at 12 h and 24 h; Table S4: GO Enrichment Analysis of Differential Spliced Genes (DSG); Table S5: KEGG Enrichment Analysis of Differentially Spliced Genes (DSG); Table S6: The expression levels of serine/arginine-rich (SR) splicing factors under cold stress; Table S7: Differentially methylated genes (DMGs) under 12 h of cold treatment; Table S8: Differentially methylated genes (DMGs) under 24 h of cold treatment.

## References

[1] Zinta G., Singh R.K., Kumar R., Satyakam (2022). Cold adaptation strategies in plants—An emerging role of epigenetics and antifreeze proteins to engineer cold resilient plants. Front. Genet..

[2] Pearce R.S. (2001). Plant Freezing and Damage. Ann. Bot..

[3] Gusain S., Joshi S., Joshi R. (2023). Sensing, signalling, and regulatory mechanism of cold-stress tolerance in plants. Plant Physiol. Biochem..

[4] Filichkin S., Priest H.D., Megraw M., Mockler T.C. (2015). Alternative splicing in plants: Directing traffic at the crossroads of adaptation and environmental stress. Curr. Opin. Plant Biol..

[5] Laloum T., Martín G., Duque P. (2018). Alternative Splicing Control of Abiotic Stress Responses. Trends Plant Sci..

[6] Shang X., Cao Y., Ma L. (2017). Alternative Splicing in Plant Genes: A Means of Regulating the Environmental Fitness of Plants. Int. J. Mol. Sci..

[7] Reddy A.S.N., Marquez Y., Kalyna M., Barta A. (2013). Complexity of the Alternative Splicing Landscape in Plants. Plant Cell.

[8] Liu Z., Qin J., Tian X., Xu S., Wang Y., Li H., Wang X., Peng H., Yao Y., Hu Z. (2018). Global profiling of alternative splicing landscape responsive to drought, heat and their combination in wheat (*Triticum aestivum* L.). Plant Biotechnol. J..

[9] Wang M., Wang P., Liang F., Ye Z., Li J., Shen C., Pei L., Wang F., Hu J., Tu L. (2017). A global survey of alternative splicing in allopolyploid cotton: Landscape, complexity and regulation. New Phytol..

[10] Zhao Y., Sun J., Xu P., Zhang R., Li L. (2014). Intron-Mediated Alternative Splicing of WOOD-ASSOCIATED NAC TRANSCRIPTION FACTOR1B Regulates Cell Wall Thickening during Fiber Development in Populus Species. Plant Physiol..

[11] Zhu G., Li W., Zhang F., Guo W. (2018). RNA-seq analysis reveals alternative splicing under salt stress in cotton, *Gossypium davidsonii*. BMC Genom..

[12] Calixto C.P.G., Guo W., James A.B., Tzioutziou N.A., Entizne J.C., Panter P.E., Knight H., Nimmo H.G., Zhang R., Brown J.W.S. (2018). Rapid and Dynamic Alternative Splicing Impacts the Arabidopsis Cold Response Transcriptome. Plant Cell.

[13] Chechanovsky N., Hovav R., Frenkel R., Faigenboim A., Eselson Y., Petreikov M., Moy M., Shen S., Schaffer A.A. (2019). Low temperature upregulates *cwp* expression and modifies alternative splicing patterns, increasing the severity of *cwp*-induced tomato fruit cuticular microfissures. Hortic. Res..

[14] Zheng Y., Luo L., Chen Q., Yang D., Gong Y., Yang Y., Qin X., Wang Y., Kong X., Yang Y. (2022). Cold Response Transcriptome Analysis of the Alternative Splicing Events Induced by the Cold Stress in D. catenatum. Int. J. Mol. Sci..

[15] Li Y., Mi X., Zhao S., Zhu J., Guo R., Xia X., Liu L., Liu S., Wei C. (2020). Comprehensive profiling of alternative splicing landscape during cold acclimation in tea plant. BMC Genom..

[16] Calixto C.P.G., Tzioutziou N.A., James A.B., Hornyik C., Guo W., Zhang R., Nimmo H.G., Brown J.W.S. (2019). Cold-Dependent Expression and Alternative Splicing of Arabidopsis Long Non-coding RNAs. Front. Plant Sci..

[17] Seo P.J., Park M.-J., Park C.-M. (2013). Alternative splicing of transcription factors in plant responses to low temperature stress: Mechanisms and functions. Planta.

[18] Egawa C., Kobayashi F., Machiko I., Nakamura T., Nakamura C., Takumi S. (2006). Differential regulation of transcript accumulation and alternative splicing of a DREB2 homolog under abiotic stress conditions in common wheat. Genes Genet. Syst..

[19] Matsukura S., Mizoi J., Yoshida T., Todaka D., Ito Y., Maruyama K., Shinozaki K., Yamaguchi-Shinozaki K. (2010). Comprehensive analysis of rice DREB2-type genes that encode transcription factors involved in the expression of abiotic stress-responsive genes. Mol. Genet. Genom..

[20] Zheng J., Wen S., Yu Z., Luo K., Rong J., Ding M. (2023). Alternative Splicing during Fiber Development in *G. hirsutum*. Int. J. Mol. Sci..

[21] Erkelenz S., Mueller I.F., Evans M.S., Busch A., Scho K., Hertel K.J., Schaal H. (2013). Position-dependent splicing activation and repression by SR and hnRNP proteins rely on common mechanisms. RNA.

[22] Chen W., Moore M.J. (2015). Spliceosomes. Curr. Biol..

[23] Koncz C., deJong F., Villacorta N., Szakonyi D., Koncz Z. (2012). The Spliceosome-Activating Complex: Molecular Mechanisms Underlying the Function of a Pleiotropic Regulator. Front. Plant Sci..

[24] Matlin A.J., Clark F., Smith C.W.J. (2005). Understanding alternative splicing: Towards a cellular code. Mol. Cell Biol..

[25] Syed N.H., Kalyna M., Marquez Y., Barta A., Brown J.W.S. (2012). Alternative splicing in plants—Coming of age. Trends Plant Sci..

[26] Palusa S.G., Ali G.S., Reddy A.S.N. (2007). Alternative splicing of pre-mRNAs of Arabidopsis serine/arginine-rich proteins: Regulation by hormones and stresses. Plant J..

[27] Zhao X., Tan L., Wang S., Shen Y., Guo L., Ye X., Liu S., Feng Y., Wu W. (2021). The SR Splicing Factors: Providing Perspectives on Their Evolution, Expression, Alternative Splicing, and Function in *Populus trichocarpa*. Int. J. Mol. Sci..

[28] Gelfman S., Cohen N., Yearim A., Ast G. (2013). DNA-methylation effect on cotranscriptional splicing is dependent on GC architecture of the exon–intron structure. Genome Res..

[29] Lev Maor G., Yearim A., Ast G. (2015). The alternative role of DNA methylation in splicing regulation. Trends Genet..

[30] Luco R.F., Allo M., Schor I.E., Kornblihtt A.R., Misteli T. (2011). Epigenetics in Alternative Pre-mRNA Splicing. Cell.

[31] Yearim A., Gelfman S., Shayevitch R., Melcer S., Glaich O., Mallm J.-P., Nissim-Rafinia M., Cohen A.-H.S., Rippe K., Meshorer E. (2015). HP1 Is Involved in Regulating the Global Impact of DNA Methylation on Alternative Splicing. Cell Rep..

[32] Zemach A., McDaniel I.E., Silva P., Zilberman D. (2010). Genome-Wide Evolutionary Analysis of Eukaryotic DNA Methylation. Sciencexpress.

[33] Li-Byarlay H., Li Y., Stroud H., Feng S., Newman T.C., Kaneda M., Hou K.K., Worley K.C., Elsik C.G., Wickline S.A. (2013). RNA interference knockdown of DNA methyl-transferase 3 affects gene alternative splicing in the honey bee. Proc. Natl. Acad. Sci. USA.

[34] Wang X., Hu L., Wang X., Li N., Xu C., Gong L., Liu B. (2016). DNA Methylation Affects Gene Alternative Splicing in Plants: An Example from Rice. Mol. Plant.

[35] Hu Y., Peng X., Wang F., Chen P., Zhao M., Shen S. (2021). Natural population re-sequencing detects the genetic basis of local adaptation to low temperature in a woody plant. Plant Mol. Biol..

[36] Peng X., Teng L., Yan X., Zhao M., Shen S. (2015). The cold responsive mechanism of the paper mulberry: Decreased photosynthesis capacity and increased starch accumulation. BMC Genom..

[37] Zhang B., Chen N., Peng X., Shen S. (2021). Identification of the PP2C gene family in paper mulberry (*Broussonetia papyrifera*) and its roles in the regulation mechanism of the response to cl4old stress. Biotechnol. Lett..

[38] Peng X., Wu Q., Teng L., Tang F., Pi Z., Shen S. (2015). Transcriptional regulation of the paper mulberry under cold stress as revealed by a comprehensive analysis of transcription factors. BMC Plant Biol..

[39] Wang A., Liu J., Huang B., Xu Y.-M., Li J., Huang L.-F., Lin J., Zhang J., Min Q.-H., Yang W.-M. (2015). Mechanism of alternative splicing and its regulation. Biomed. Rep..

[40] Robin Lytle J., Steitz J.A. (2004). Premature termination codons do not affect the rate of splicing of neighboring introns. RNA.

[41] Thatcher S.R., Danilevskaya O.N., Meng X., Beatty M., Zastrow-Hayes G., Harris C., Allen B.V., Habben J., Li B. (2016). Genome-wide analysis of alternative splicing during development and drought stress in maize. Plant Physiol..

[42] Li S., Yu X., Cheng Z., Zeng C., Li W., Zhang L., Peng M. (2020). Large-scale analysis of the cassava transcriptome reveals the impact of cold stress on alternative splicing. J. Exp. Bot..

[43] Shen Y., Zhou Z., Wang Z., Li W., Fang C., Wu M., Ma Y., Liu T., Kong L.-A., Peng D.-L. (2014). Global Dissection of Alternative Splicing in Paleopolyploid Soybean. Plant Cell.

[44] Yang J., Chen A., Lv W., Shao L., Fu Y., Liu H., Xie X., Wang Z., Li C. (2021). PacBio and Illumina RNA Sequencin Identify Alternative Splicing Events in Response to Cold Stress in Two Poplar Species. Front. Plant Sci..

[45] Li M., Ou M., He X., Ye H., Ma J., Liu H., Yang H., Zhao P. (2023). DNA methylation role in subgenome expression dominance of *Juglans regia* and its wild relative *J. mandshurica*. Plant Physiol..

[46] Stark R., Grzelak M., Hadfield J. (2019). RNA sequencing: The teenage years. Nat. Rev. Genet..

[47] Chen S., Zhou Y., Chen Y., Gu J. (2018). fastp: An ultra-fast all-in-one FASTQ preprocessor. Bioinformatics.

[48] Kim D., Paggi J.M., Park C., Bennett C., Salzberg S.L. (2019). Graph-based genome alignment and genotyping with HISAT2 and HISAT-genotype. Nat. Biotechnol..

[49] Pertea G., Pertea M. (2020). GFF Utilities: GffRead and GffCompare [version 1; peer review: 3 approved]. F1000Research.

[50] Liao Y., Smyth G.K., Shi W. (2019). The R package Rsubreadis easier, faster, cheaper and better for alignment and quantification of RNA sequencing reads. Nucleic Acids Res..

[51] Pertea M., Kim D., Pertea G.M., Leek J.T., Salzberg S.L. (2016). Transcript-level expression analysis of RNA-seq experiments with HISAT, StringTie and Ballgown. Nat. Protoc..

[52] Patro R., Duggal G., Love M.I., Irizarry R.A., Kingsford C. (2017). Salmon provides fast and bias-aware quantification of transcript expression. Nat. Methods.

[53] Grabherr M.G., Haas B.J., Yassour M., Levin J.Z., Thompson D.A., Amit I., Adiconis X., Fan L., Raychowdhury R., Zeng Q. (2011). Full-length transcriptome assembly from RNA-Seq data without a reference genome. Nat. Biotechnol..

[54] Trincado J.L., Entizne J.C., Hysenaj G., Singh B., Skalic M., Elliott D.J., Eyras E. (2018). SUPPA2: Fast, accurate, and uncertaintyaware differential splicing analysis across multiple conditions. Genome Biol..

[55] Cantalapiedra C.P., Hernández-Plaza A., Letunic I., Bork P., Huerta-Cepas J. (2021). eggNOG-mapper v2: Functional Annotation, Orthology Assignments, and Domain Prediction at the Metagenomic Scale. Mol. Biol. Evol..

[56] Aramaki T., Blanc-Mathieu R., Endo H., Ohkubo K., Kanehisa M., Goto S., Ogata H. (2020). KofamKOALA: KEGG Ortholog assignment based on profile HMM and adaptive score threshold. Bioinformatics.

[57] Katoh K., Standley D.M. (2013). MAFFT Multiple Sequence Alignment Software Version7Improvements in Performance and Usability. Mol. Biol. Evol..

[58] Eddy S.R. (2011). Accelerated Profile HMM Searches. PLoS Comput. Biol..

[59] Krueger F., Andrews S.R. (2011). Bismark: A flexible aligner and methylation caller for Bisulfite-Seq applications. Bioinformatics.

[60] Zhou Q., Lim J.-Q., Sung W.-K., Li G. (2019). An integrated package for bisulfite DNA methylation data analysis with Indelsensitive mapping. BMC Bioinform..

[61] Akalin A., Kormaksson M., Li S., Garrett-Bakelman F.E., Figueroa M.E., Melnick A., Mason C.E. (2012). methylKit: A comprehensive R package for the analysis of genome-wide DNA methylation profiles. Genome Biol..

